# TMED2 promotes epithelial ovarian cancer growth

**DOI:** 10.18632/oncotarget.21593

**Published:** 2017-10-06

**Authors:** Gong Shi-Peng, Chen Chun-Lin, Wu Huan, Meng Fan-Liang, Chen Yong-Ning, Zhang Ya-Di, Zhang Guang-Ping, Cai Ye-Ping

**Affiliations:** ^1^ Department of Obstetrics and Gynecology, Nanfang Hospital, Southern Medical University, Guangzhou 510515, P.R. China; ^2^ Department of Obstetrics and Gynecology, The Second Affiliated Hospital, Chongqing University of Medical Sciences, Chongqing 400010, P.R. China; ^3^ Department of Gynecology, People's Hospital of Huadu District, Guangzhou 510800, P.R. China; ^4^ Department of Gynecology, Maternal and Child Health Hospital of Duanzhou District, Zhaoqing 526000, P.R. China

**Keywords:** epithelial ovarian cancer, TMED2, IGF1R, AKT, miR-30a

## Abstract

TMED2 is involved in morphogenesis of the mouse embryo and placenta. We found that expression of TMED2 was higher in epithelial ovarian cancer tissues than normal ovarian tissues. Silencing TMED2 decreased cell proliferation, migration, and invasion. Ectopic expression of TMED2 increased cell proliferation, migration and invasion. Silencing TMED2 inhibited ovarian cancer growth in mice. Silencing TMED2 inhibited IGF2/IGF1R/PI3K/Akt pathway. In agreement, ectopically expressed TMED2 activated IGF2/IGF1R/PI3K/Akt pathway. Mechanistic study revealed that TMED2 directly binds to AKT2, thereby facilitating its phosphorylation. We also found that TMED2 increased IGF1R expression by competing for miR-30a. Thus, TMED2 is oncogenic and a potential target for epithelial ovarian cancer therapy.

## INTRODUCTION

TMED2 is a member of the tranmembrane emp24 domain [1, 2]. TMED2 was involved in the development of mouse embryos [3]. Previous study reported that ectopic expression of TMED2 can accelerate the proliferation in MC3T3-E1 cell [4]. This indicated its possible role in cancer cells proliferation. However, the role of TMED2 in epithelial ovarian carcinoma is still unknown. IGF1R is a transmembrane tyrosine kinase. It is often increased expression in many cancers. It promotes proliferation and inhibits apoptosis. IGF signaling pathway also mediates protease secretion, hypoxia signaling, cancer cell motility and adhesion. So, it is related to the invasion and metastasis in numerous cancers. Therefore, the IGF1R is a potential anti-cancer target. In some preclinical models of cancer, IGF1R antibody is showing promise [5, 6]. Activate IGF2/PI3K/AKT signaling pathway promotes glioblastoma multiforme progression [7]. However, the role of TMED2 in IGF2/IGF1R/PI3K/AKT pathway is not elucidated yet.

In this research, we noted an elevated expression of TMED2 in epithelial ovarian carcinoma tissues. Our data also found that TMED2 promoted the ability of proliferation, migration, invasion in ovarian cancer cells. we also observed TMED2 can modulate IGF2/IGF1R/ PI3K/AKT pathway. Further, we identified TMED2 directly binds to AKT2, thereby facilitating its phosphorylation. TMED2 served as a competing endogenous RNA (ceRNA) to regulate the expression of IGF1R through competing for miR-30a.

## RESULTS

### The expression of TMED2 was increased in epithelial ovarian cancer

**Figure 1:**
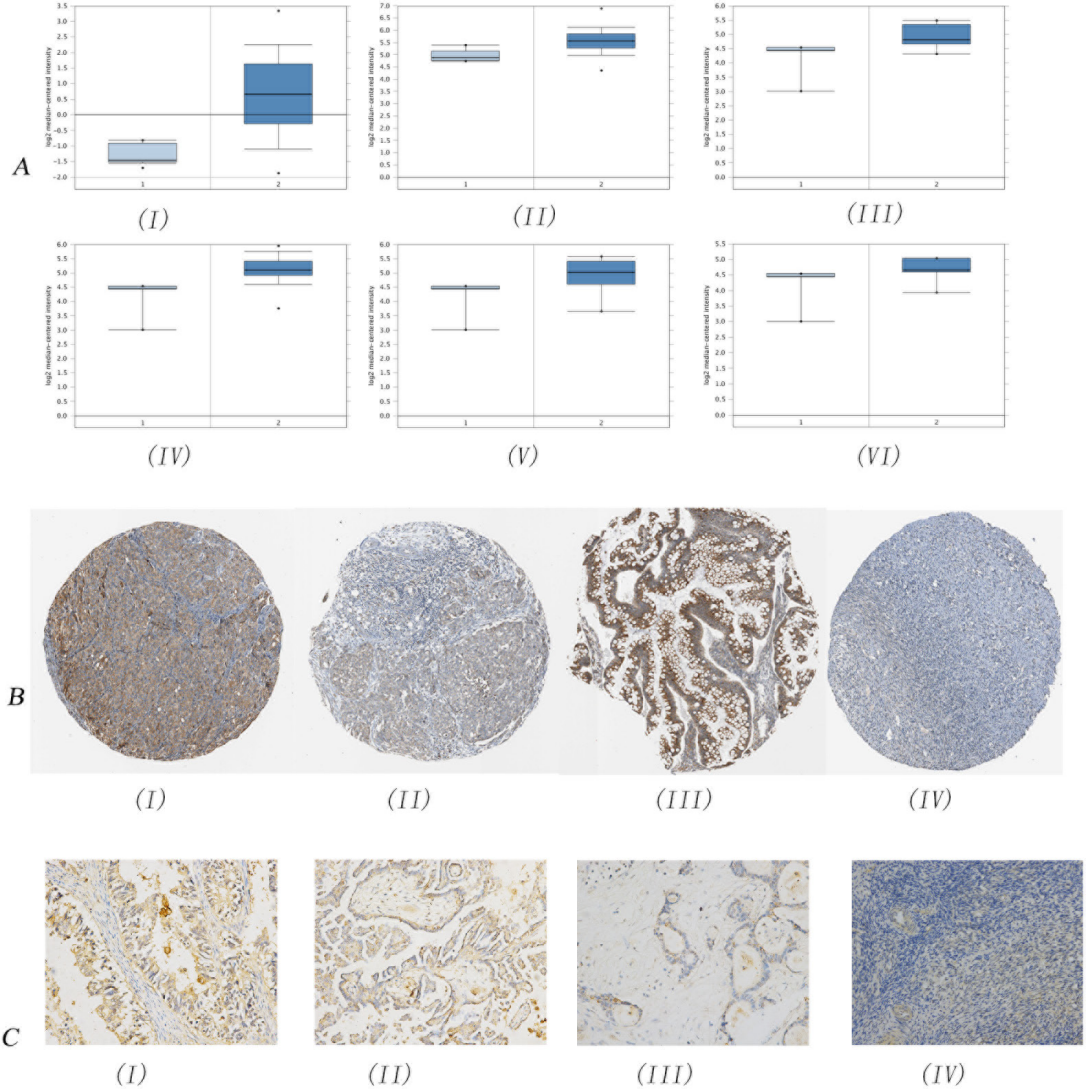
Immunohistochemical analysis of TMED2 expression in ovarian cancer The mRNA expression of TMED2 in ovarian cancer was analyzed. Data derived from Oncomine database. The mRNA expression of TMED2 in ovarian cancer was increased compared to normal ovarian tissues **(A(I-II))**. The expression of TMED2 was increased in ovarian mucinous adenocarcinoma, ovarian serous adenocarcinoma and ovarian endometrioid adenocarcinoma was elevated compared to normal ovarian tissues (AIII-V). TMED2 was located in cytoplasmic and membranous **(B)**. The TMED2 in ovarian mucinous adenocarcinoma, ovarian serous adenocarcinoma and ovarian endometrioid adenocarcinoma was moderate expression(BI-III). The expression of TMED2 was determined in endometrioid adenocarcinoma **(CI)**, serous papillary adenocarcinoma (CII), mucinous adenocarcinoma (CIII) and normal ovarian tissues (CIV). Original magnification, 200X.

We firstly analyzed the mRNA expression of TMED2 in ovarian carcinoma derived from Oncomine database. As showed in Figure 1A(I), the mRNA expression of TMED2 in ovarian carcinoma was increased compared with normal ovarian tissues(P=8.91E-16). The expression of TMED2 was increased in ovarian serous cystadenocarcinoma compared with normal ovarian tissues(Figure 1A(II); P=5.21E-5). The expression of TMED2 was increased in ovarian mucinous adenocarcinoma, ovarian serous adenocarcinoma and ovarian endometrioid adenocarcinoma was elevated compared to normal ovarian tissues(Figure 1A(III-V); P=0.043, 0.016 and 0.049 respectively). However, the expression difference of TMED2 between ovarian clear cell adenocarcinoma and normal ovarian tissues is not significant(P=0.102). We next analyzed the expression and location of TMED2 in ovarian carcinoma tissues derived from Human Protein Atlas. As showed in Figure 1B, TMED2 was located in cytoplasmic and membranous. The TMED2 in ovarian mucinous adenocarcinoma, ovarian serous adenocarcinoma and ovarian endometrioid adenocarcinoma was moderate expression(Figure 1B(I-III)). The TMED2 in follicle cells was medium expression. However, the TMED2 was not detected in ovarian stroma cells(Figure 1B(IV)).

We then determined the expression and location of TMED2 in epithelial ovarian carcinoma tissues and normal ovarian tissues. The expression of TMED2 was elevated in epithelial ovarian carcinoma tissues compared to that in normal ovarian tissues(Figure 1C). TMED2 was mainly localized on the cell membrane and cytoplasm (Figure 1C(I-III)). In this research, we found an increased expression of TMED2 in epithelial ovarian carcinoma tissues compared to that in normal ovary tissues (P<0.05; Figure 1C and Table 1). Then, we determined the relationship between TMED2 expression and clinicopathologic variables of 148 epithelial ovarian carcinoma tissues (Table 1). Significantly higher expression of TMED2 was observed in advanced stages than those in the early stages (P<0.05). Further, the expression of TMED2 was related to the cancer grade(P<0.05). However, there was no significant correlation between TMED2 expression and age(P>0.05; Table 1).

**Table 1:** Association of TMED2 expression with clinicopathological characteristics in 148 patients of EOC

	No. of patients	TMED2 expression		P value
	(n=148)	Low no.(%)	High no.(%)	
Characteristics				
Age(years)				>0.05
<50	78	30(38.46%)	48(61.54%)	
≥50	70	33(47.14%)	37(52.86%)	
Normal ovarian	20	16(80.00%)	4(20.00%)	<0.05
Cancer tissues	128	47(36.72)	81(63.28%)	
FIGO stage				
I/II	98	6162.24%)	37(37.76%)	<0.05
III/IV	30	2(6.67%)	28(93.33%)	
Grade				
1	28	23(82.14%)	5(17.86%)	
2	34	22(64.71%)	12(35.29%)	
3	66	24(36.36%) Grade 2-3 versus 1	42(63.64%)	<0.05

### TMED2 regulates cellular proliferation

**Figure 2:**
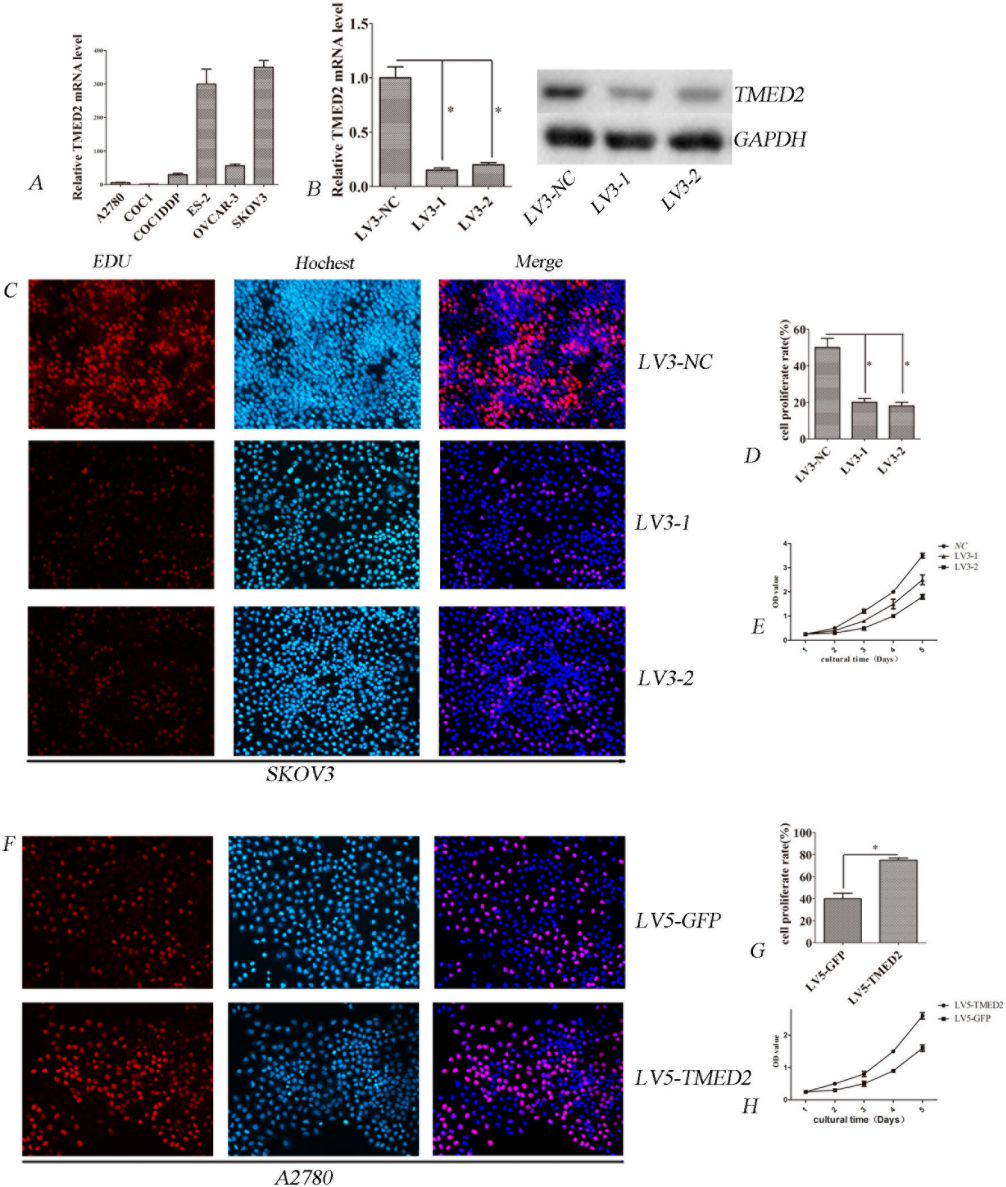
TMED2 regulates cellular proliferation **(A)** The relative expression of TMED2 mRNA in ovarian cancer cell lines. **(B)** TMED2 mRNA and protein level were down-regulated by infected with LV3-1 or LV3-2. **(C, D)** Ovarian cancer SKOV3 cells were infected with LV3-NC, LV3-1 and LV3-2. Cell proliferation was assessed by EdU. The proliferation rate of LV3-1 and LV3-2 cells was lower than that of LV3-NC cells. Original magnification, 200X. **(E)** Cell proliferation was determined by CCK-8 assay. **(F, G)** Ovarian cancer A2780 cells were infected with LV5-GFP or LV5-TMED2. Then cell proliferation was determined by EdU. Original magnification, 200X. The proliferation rate of LV5-TMED2 cells was increased compared with LV5-GFP cells. **(H)** Cell proliferation was determined by CCK-8 assay. Error bars represent standard error. ^*^ p<0.05, and ^**^p<0.001.

We firstly investigated the mRNA level of TMED2 in six ovarian cancer cell lines using qPCR. We found that TMED2 was up-regulated in SKOV3 and ES-2 cell lines. At the same time, TMED2 was down-regulated in A2780 cell line(Figure 2A). Thus, SKOV3 cells were used to silence TMED2 expression, A2780 cells were used for ectopic expression of TMED2. To study the function of TMED2 in SKOV3 cells, we silenced the expression of the TMED2 gene. The expression of TMED2 was decreased after infection of LV3-1 or LV3-2 in SKOV3 cells (Figure 2A and 2B). We found that down-regulation of TMED2 after infection of LV3-1 and LV3-2 suppressed cell proliferation in SKOV3 cells (P < 0.05; Figure 2C, 2D and 2E). We also found that the proliferation ability was higher in LV5-TMED2 infected A2780 cells than that in LV-5-GFP-infected cells (P<0.05; Figure 2F, 2G and 2H).

### TMED2 regulated cellular migration, invasion *in vitro*

**Figure 3:**
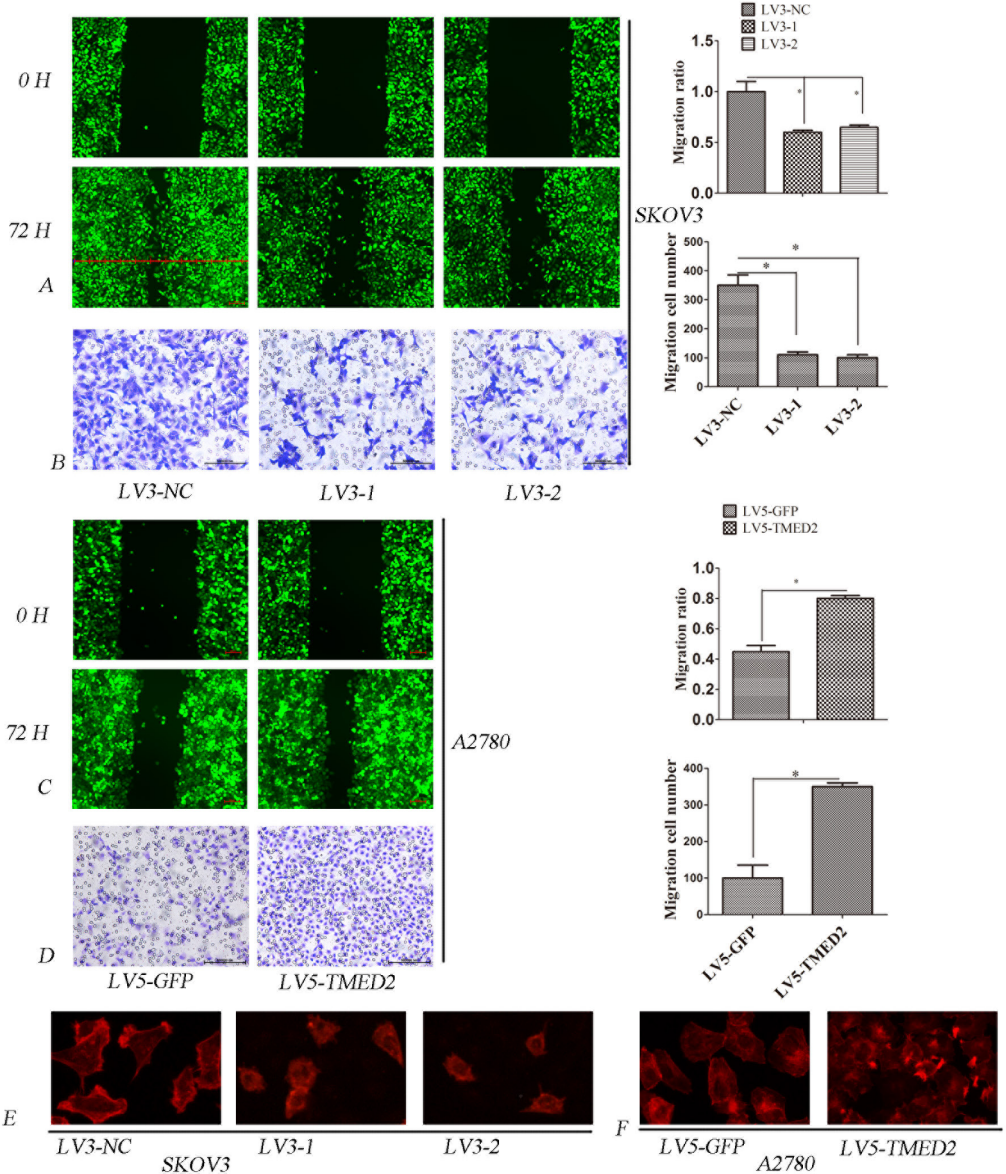
**(A)** Ovarian cancer SKOV3 cells migration ability were detected by the wound healing assay. The migration of LV3-1 and LV3-2 infected SKOV3 cells was lower as compared with LV3-NC infected cells. **(B)** Ovarian cancer SKOV3 cells invasion ability was detected by Matrigel invasion assays. The invasion ability of LV3-1 and LV3-2 infected SKOV3 cells was decreased compared with LV3-NC infected cells. **(C)** Ovarian cancer A2780 cells migration ability was detected by wound healing assay. The migration ability of LV5-TMED2 infected A2780 cells was increased compared with LV3-NC infected cells. **(D)** Ovarian cancer A2780 cells invasion ability was detected by Matrigel invasion assays. The invasion ability of LV5-TMED2 infected A2780 cells was increased compared with LV5-GFP infected cells. **(E** and **F)** F-actin staining. Original magnification, 400X. Error bars represent standard error. ^*^ p<0.05, and ^**^p<0.001.

In order to determined the role of TMED2 in cell metastasis and invasion, wound healing assay and matrigel invasion assay were performed. Our results showed that the migration and invasion ability of LV3-1 or LV3-2 infected SKOV3 cells were reduced compared to that of LV3-NC infected SKOV3 cells(p < 0.05) (Figure 3A and 3B). However, the migration and invasion ability of LV5-TMED2 infected A2780 cells were increased compared to that of LV5-GFP infected A2780 cells (P < 0.05)(Figure 3C and 3D).

Cytoskeleton plays a very important role in the invasion and metastasis of tumor cells [8]. Therefore, we determined whether TMED2 regulated the cytoskeleton rearrangement using phalloidin staining. We found that F-actin was mostly localized in the cellular projections and outgrowth. Silencing TMED2 expression in SKOV3 cells, the formation of lamellipodia and membrane ruffles was suppressed(Figure 3E). A2780 cells infection of LV5-GFP showed some small ruffles. However, ectopic expression of TMED2 in A2780 cells induced F-actin reorganization at lamellipodia and membrane ruffles(Figure 3F).

### TMED2 regulation of IGF1R is miR-30a dependent

**Figure 4:**
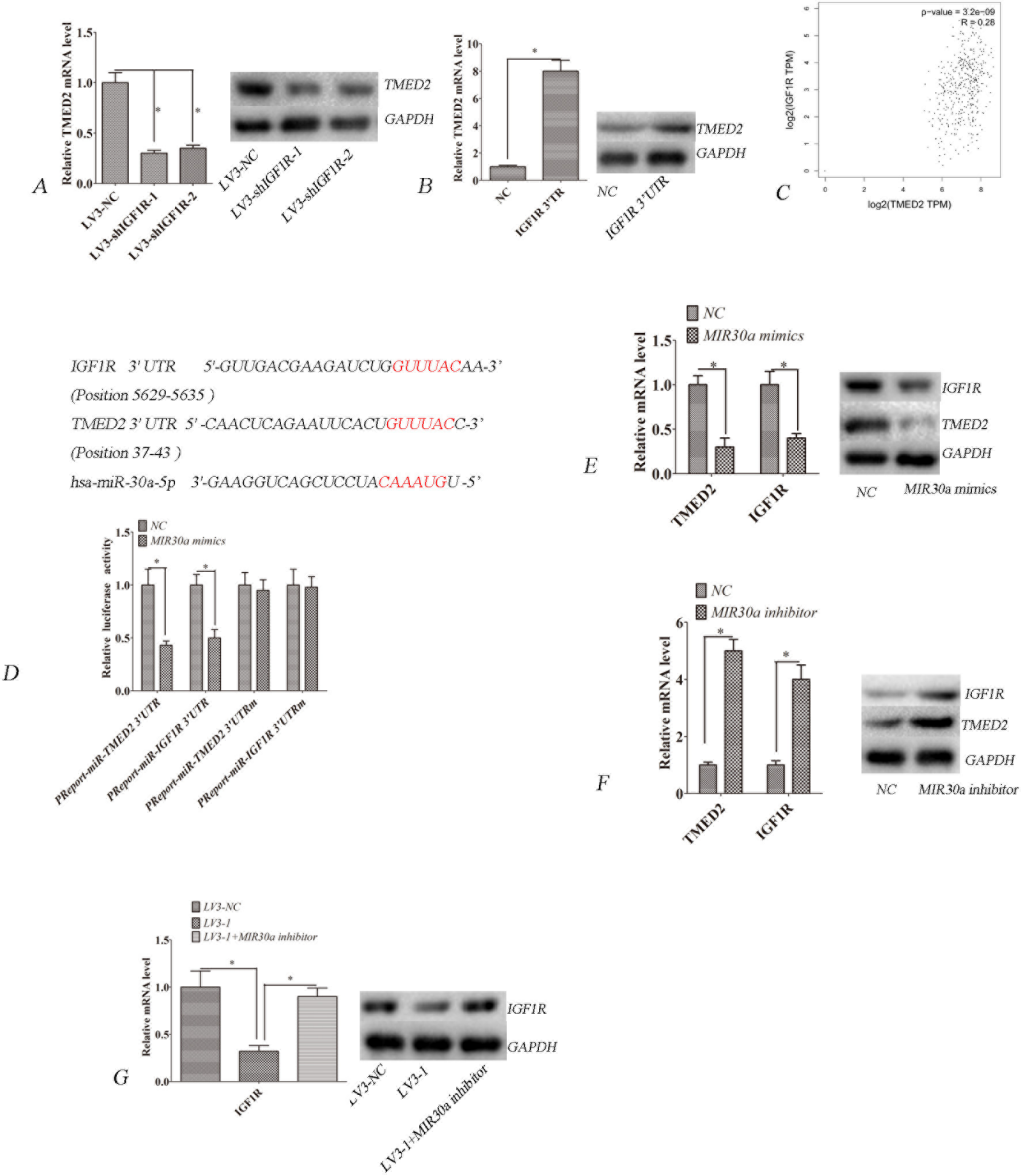
TMED2 regulates IGF1R mRNA and protein levels **(A)** TMED2 mRNA and protein levels were downregulated by infection with LV3-shIGF1R-1 and LV3- shIGF1R-2. **(B)** Ovarian cancer SKOV3 cells were transfected with IGF1R 3'UTR and negative control. TMED2 mRNA and protein levels were increased by transfection with IGF1R 3'UTR. **(C)** The expression of TMED2 in ovarian cancer was positively correlated with IGF1R. Data derived from the The TCGA database (R=0.28; P=3.2E-09; http://gepia.cancer-pku.cn/detail.php). **(D)** Putative binding sites targeted by miR-30a were predicted to be located in the 3' UTR of TMED2 and IGF1R mRNA. SKOV3 cells were cotransfected with miR-30a mimics or control RNA (NC) with luciferase reporter plasmids containing either wild-type (pMIR-TMED2-3UTR and pMIR-IGF1R-3UTR) or mutant 3' UTR (pMIR-TMED2-3UTRm and pMIR-IGF1R-3UTRm) of TMED2 and IGF1R genes. Luciferase expression was measured. The fold changes of the relative luciferase activity in miR-30a mimics with the indicated plasmids transfected cells were normalized to NC with the corresponding indicated plasmid-transfected cells. **(E)** Ovarian cancer SKOV3 cells were transfected with miR-30a mimics or control RNA (NC). **(F)** Ovarian cancer SKOV3 cells were transfected with miR-30a inhibitor or control RNA (NC). **(G)** Ovarian cancer SKOV3 cells were infected with LV3-NC and LV3-1. One group was infected with LV3-1-transfected miR-30a inhibitors. Error bars represent standard error. The symbols ^*^ and ^**^ indicate p < 0.05 and 0.01, respectively.

Bioinformatics analyses (Targetscan) showed that IGF1R is a putative ceRNA of TMED2. We observed that IGF1R mRNA and protein expression were obviously reduced in LV3-shIGF1R-1 or LV3-shIGF1R-2 infected SKOV3 cells compared to LV3-NC infected SKOV3 cells (Figure 4A; P<0.05). However, the mRNA and protein expression of IGF1R were upregulated when the TMED2 3' untranslated region (UTR) was ectopically expressed in A2780 cells (Figure 4B; P<0.05). The TCGA data indicated that the expression of TMED2 in ovarian cancer was positively correlated with IGF1R (Figure 4C, R=0.28; P=3.2E-09)(http://gepia.cancer-pku.cn/detail.php)[9].

We investigated whether the effect of TMED2 on IGF1R is miRNA dependent. Interestingly, bioinformatics analyses showed that TMED2 and IGF1R are predictive targets of miR-30a (Targetscan). Thus, we attempted to experimentally verify whether miR-30a modulated TMED2 and IGF1R expressions in SKOV3 cells. Bioinformatics predicted that there was a miR-30a binding site in the 3' UTR of TMED2 and IGF1R mRNAs (Figure 4D). We constructed a vector to determine whether miR-30a can directly target the 3' UTR of TMED2 and IGF1R. Our results showed that miR-30a mimics significantly suppressed luciferase activity of the reporter vector. In contrast, no significant suppression of luciferase activity was detected in cells transfected with the control vector with mutant TMED2 and IGF1R 3' UTR when miR-30a expression was elevated (Figure 4D; P<0.05). miR-30a mimics could reduce the expression of TMED2 and IGF1R (Figure 4E; P<0.05), whereas miR-30a inhibitor could elevate the expression of these genes (Figure 4F; P<0.05). These data indicate that TMED2 and IGF1R are direct targets of miR-30a. Furthermore, we found that TMED2's regulation of IGF1R was eliminated when miR-30a was inhibited (Figure 4G; P<0.05). These data indicate that TMED2's regulation of IGF1R is miR-30a dependent.

### TMED2 regulated IGF1R/IGF2/PI3K/AKT pathway

**Figure 5:**
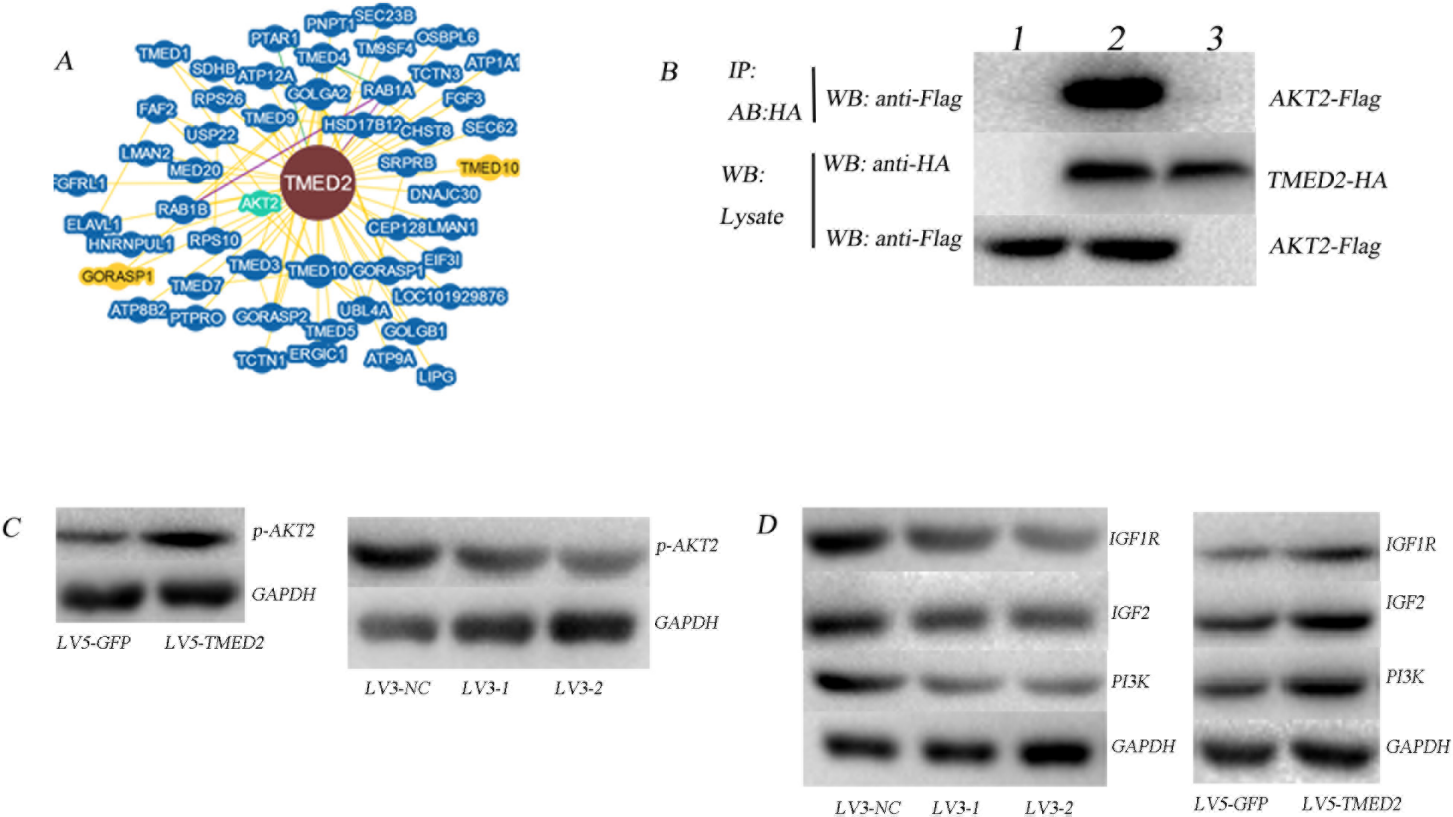
**(A)** Bioinformatics analyses showed that TMED2 may interacted with AKT2 (https://thebiogrid.org/). **(B)** The interaction between TMED2 and AKT2 was identified by Co-IP assay. Cells were co-transfected with Flag-AKT2 and HA-TMED2, and control group was established simultaneously, cells were then harvested 24 h later. Anti-HA antibodies were used to pull the interaction protein. Then, they were detected by anti-Flag antibodies. Results showed that Flag bands could not be detected in the cells transfected with Flag-AKT2 (lane 3) or HA- TMED2 (lane 1) only. However, it can be detected in cells co-transfected with both Flag-AKT2 and HA-TMED2 (lane 2), which indicated that there existed interaction between TMED2 and AKT2 *in vivo*. **(C-D)** The expression of P-AKT2, IGF1R, IGF2 and PI3K was determined by Western Blotting.

Bioinformatics analyses (https://thebiogrid.org/) showed that TMED2 may interacted with AKT2(Figure 5A). In order to identify the interaction between TMED2 and AKT2, we designed a Co-immunoprecipitation (Co-IP) experiment. Cells were co-transfected with Flag- AKT2 and HA- TMED2. Control group was established simultaneously, and then were harvested 24 h later. Anti-HA antibodies were used to pull the interaction protein. Then, they were detected by anti-Flag antibodies using Western blot. Our results indicated that Flag bands could not be detected in cells infected with Flag-AKT2 or HA-TMED2 only. However, It can be detected in cells that co-transfected with Flag-AKT2 and HA-TMED2(Figure 5B). We also observed that overexpression of TMED2 increased the expression of p-AKT2; however, silencing TMED2 decreased the expression of p-AKT2(Figure 5C). This data suggested that TMED2 directly binds to AKT and promoted its phosphorylation.

Previous studies suggested that IGF2 participate in the malignant behavior of ovarian cancer through PI3K/Akt signal pathway [10, 11]. Based on the above results, we guessed TMED2 may regulated the IGF1R/IGF2/PI3K/AKT pathway. The expression of IGF1R, IGF2, PI3K and p-AKT was reduced in SKOV3 cells infected with LV3-1 or LV3-2. In contrast, the expression of IGF1R, IGF2, PI3K and p-AKT was increased in A2780 cells infected with LV5-TMED2 (Figure 5D).

### TMED2 promoted A2780 cellular proliferation, migration and invasion involves in IGF1R/IGF2/PI3K/AKT pathway

**Figure 6:**
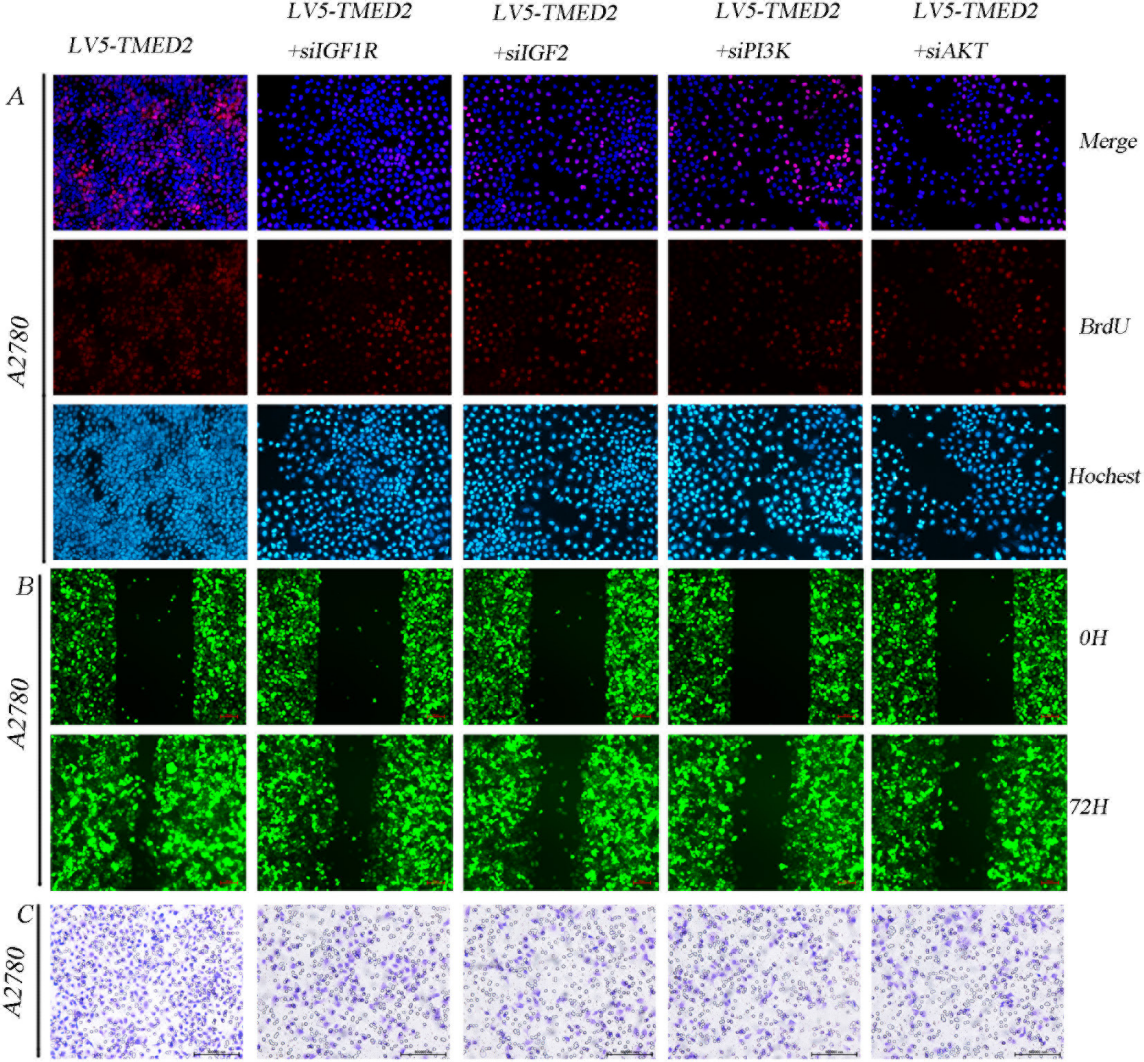
TMED2 promoted A2780 cellular proliferation, migration and invasion involves IGF1R/IGF2/PI3K/AKT pathway A2780 cells infected with LV5-GFP and LV5-TMED2. After 48 h, puromycin was added at a concentration of 2.5 μg/ml. The cells were transfected with IGF1R siRNA, IGF2 siRNA, PI3K siRNA or AKT siRNA for 48 h. Then the cellular proliferation ability **(A)**, migration ability **(B)** and invasion ability **(C)** were detected. Original magnification, 200X.

We have demonstrated that TMED2 can regulate the IGF1R/IGF2/PI3K/AKT signaling axis. Hence, we presumed that TMED2 regulation of epithelial ovarian cancer proliferation, migration and invasion involves IGF1R/IGF2/PI3K/AKT pathway. Our data showed that the ability of cellular proliferation, migration and invasion was elevated in A2780 cells infected with LV5-TMED2 compared to A2780 cells infected with LV5-GFP. Silencing of IGF1R, IGF2, PI3K or AKT partially abrogated this effect of TMED2 overexpression (Figure 6A-6C).

### miR-30a regulated SKOV3 cellular proliferation, migration and invasion through directly targeting TMED2

**Figure 7:**
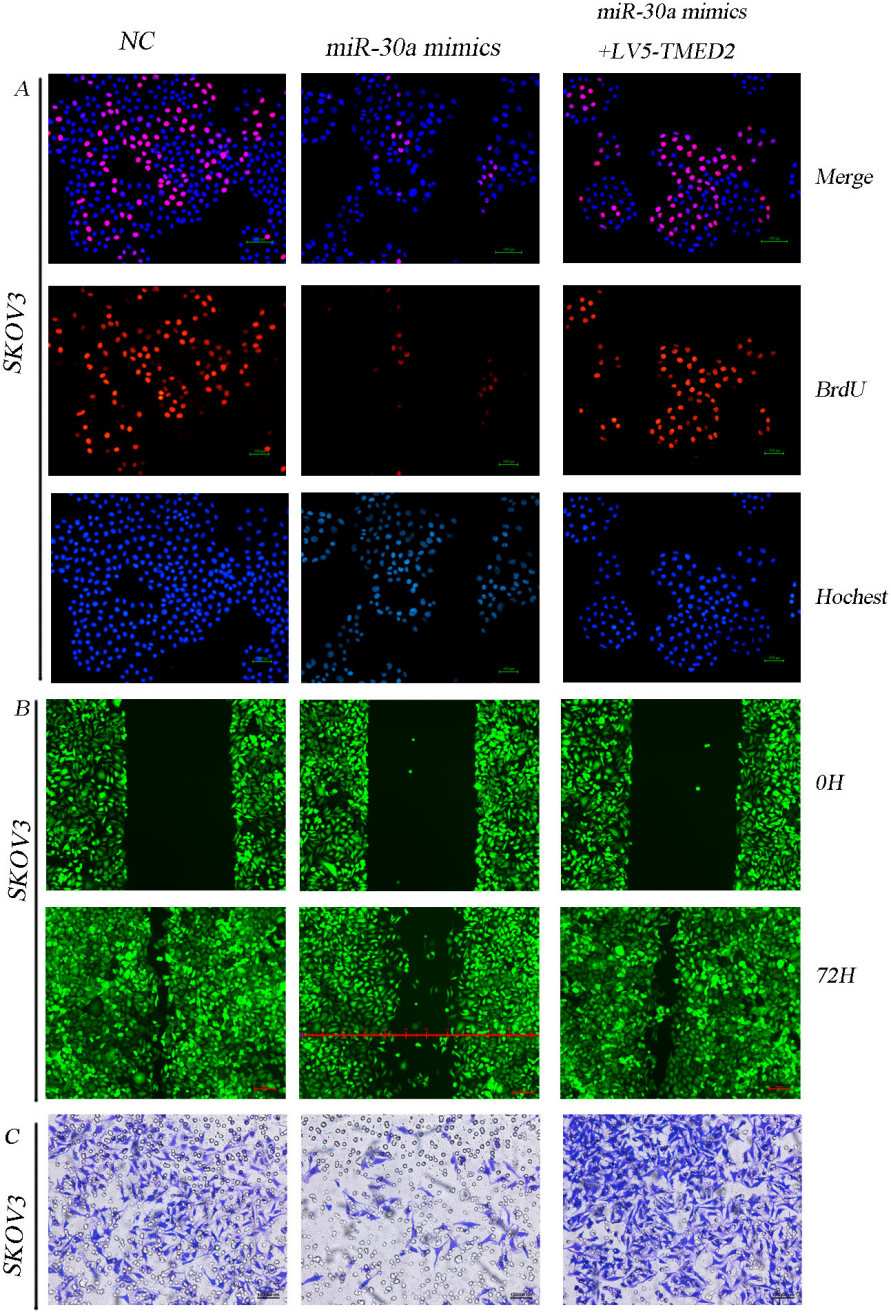
miR-30a regulated SKOV3 cellular proliferation, migration and invasion through directly targeting TMED2 Ovarian cancer SKOV3 cells were transfected with NC(negative control), miR-30a mimics or LV5-TMED2 + miR-30a mimics. Then the cellular proliferation ability **(A)**, migration ability **(B)** and invasion ability **(C)** were detected. Original magnification, 200X.

We have demonstrated TMED2 is a directly target. So, we investigated whether miR-30a regulated the malignant behavior through targeting TMED2 in SKOV3 cells. miR-30a mimics inhibited the malignant behavior in ovarian carcinoma SKOV3 cells. Ectopic expression of TMED2 can partially restored those phenotypes (Figure 7A-7C).

### Silencing of TMED2 retarded the growth of ovarian cancer SKOV3 cells *in vivo*

**Figure 8:**
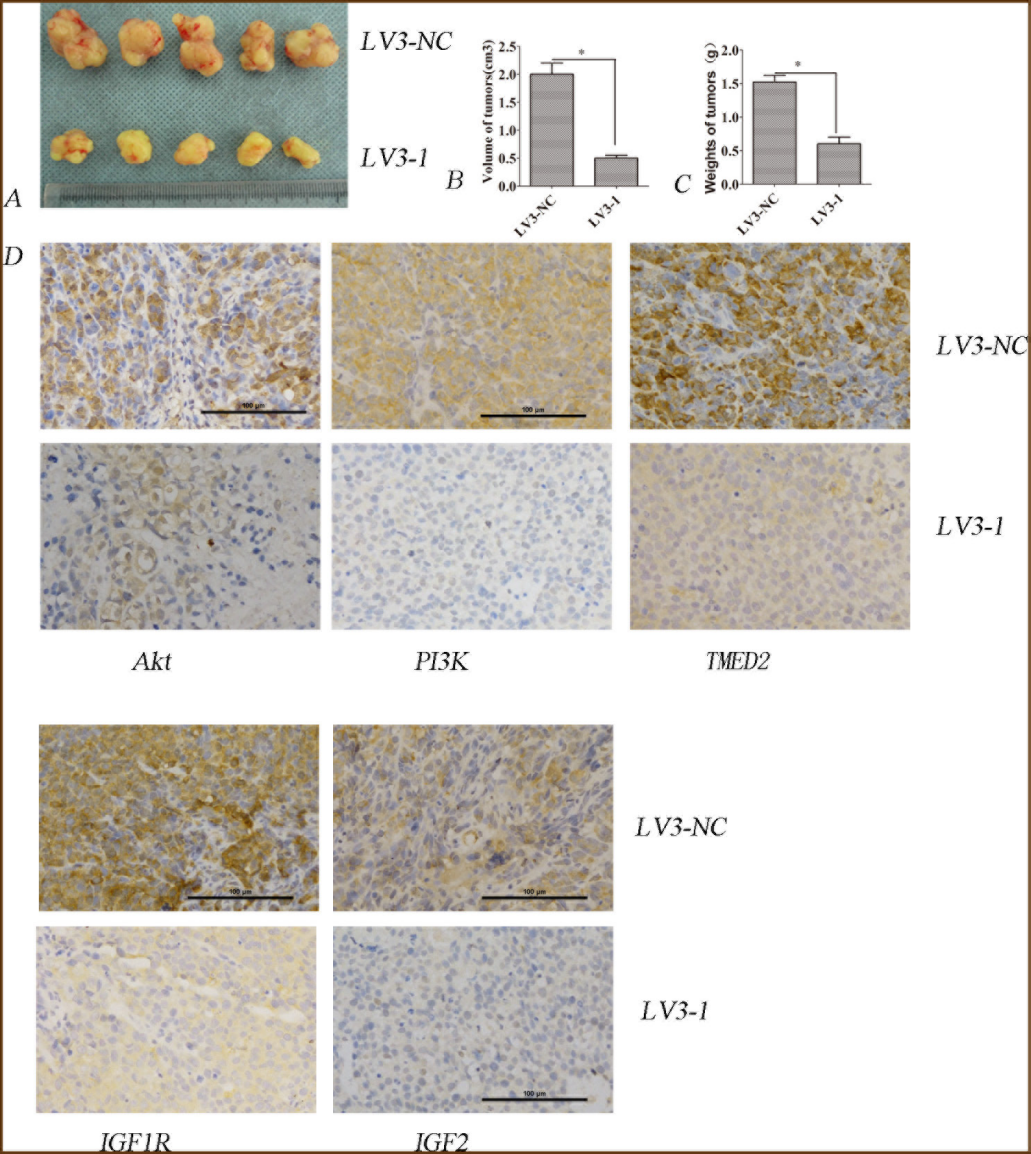
TMED2 regulated tumorigenesis in nude mice model (**A**, **B** and **C**) Mean tumor volume and weight on day 28 after tumor cell injection. LV3-1 or LV3-NC infected SKOV3 cells were implanted s.c. into the left armpit. **(D)**. Immunohistochemical analysis of TMED2, IGF1R, IGF2, PI3K or p-AKT expression were performed on tumor xenografts. Representative images are shown (original magnification ×200). ^*^ p<0.05, and ^**^p<0.001.

The regulation of TMED2 in the growth of ovarian cancer SKOV3 cells *in vivo* was determined using tumor xenografts model. The average volume of tumors was reduced in SKOV3 cells infected with LV3-1 compared to that of SKOV3 cells infected with LV3-NC (P<0.05) (Figure 8A and 8B). The average weight of tumors in SKOV3 cells infected with LV3-1 was reduced compared to that of SKOV3 cells infected with LV3-NC (P<0.05; Figure 8C). The expression of TMED2, IGF1R, IGF2, PI3K or p-AKT in tumors from SKOV3 cells was reduced when silencing TMED2 (P<0.05; Figure 8D).

## DISCUSSION

**Figure 9:**
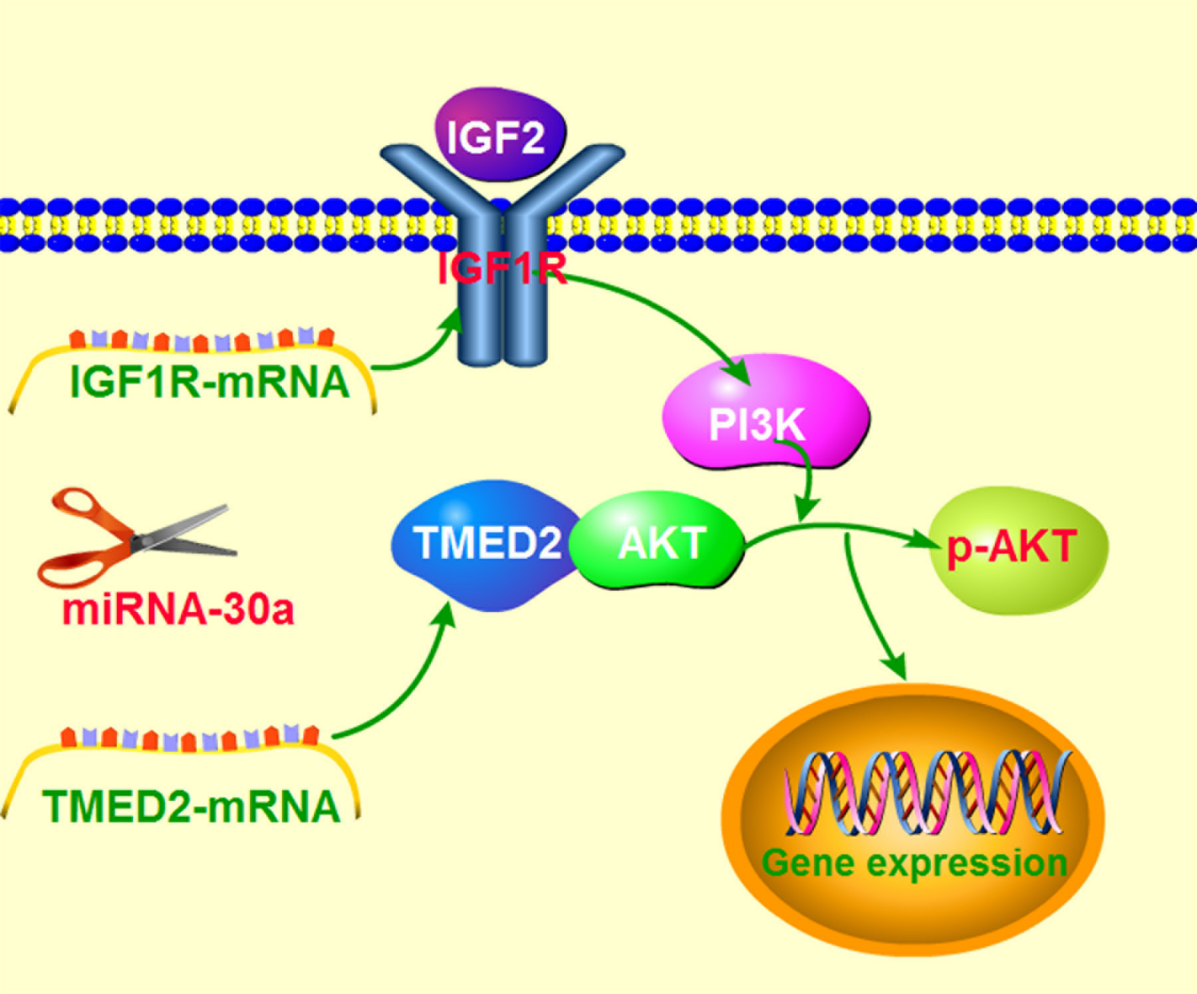
Schematic model demonstrating TMED2 function in ovarian cancer and potential mechanisms

In our research, we observed an increased expression of TMED2 in epithelial ovarian carcinoma compared with the normal ovarian tissues. The high expression of TMED2 in epithelial ovarian cancer was related to the histological grades and cancer stage. We also found that the elevated expression of TMED2 in epithelial ovarian carcinoma promoted proliferation and invasion. Our results also demonstrate that TMED2 can regulate IGF1R in a miR-30a-dependent manner (Figure 9). Based on these findings, TMED2 can serve as a new ovarian cancer biomarkers and a potential cancer treatment target.

Competing endogenous RNAs (ceRNAs) is a hypothesis that transcripts regulate each other by competing for the same miRNAs [12]. CeRNA-like regulation, including regulators, targets, modulators that can be non-coding RNAs, proteins, and even DNA regions, can regulate gene expression [13]. The ceRNA effect consists of a positive effective interaction that can arise between transcripts that are targeted by the same miRNA species due to competition [14]. Many studies suggested that ceRNAs were involved in a lot of biological processes, such as post-transcriptional regulation of gene expression [15]. To explore the functions of TMED2 in epithelial ovarian cancer, we predicted the candidate ceRNAs of TMED2 by bioinformatics analyses [16]. Our results indicate that TMED2 and IGF1R regulate each other through competition for miR-30a. We also found that the expression of TMED2 was correlated with IGF1R.

IGF1R is a transmembrane tyrosine kinase which is usually increased expression in several caner types. It promotes cellular proliferation and inhibits apoptosis in cancer cells. IGF participates in many signal pathway including PI3K-AKT pathway, cAMP-PKA pathway and MEK-ERK1/2 pathway. Therefore, the IGF1R is a potential cancer treatment target [5, 6]. Our data showed that TMED2 is a ceRNA of IGF1R. We also demonstrated that silencing IGF1R can partly inhibited the malignant behavior of A2780 cells induced through ectopic expression of TMED2. So we assumed that inhibited the IGF1R pathway may be a potential target of patients who over-expression of TMED2.

AKT is at the hub of some signal pathway. It promotes the malignant behavior of cancer [17]. A previous study demonstrated that AKT was up-regulated in ovarian cancer tissues and cell lines [18]. Inhibition of AKT activation sensitized cell survival in cisplatin resistant epithelial ovarian cancer [19, 20]. Our data indicated that TMED2 directly binds to AKT2, thereby facilitating its phosphorylation. So the over-expression of TMED2 maybe promote malignant behavior of ovarian cancer by activating AKT pathway. Activating IGF2/PI3K/AKT pathway promotes glioblastomas multiforme progression [7]. Our study found that TMED2 regulates epithelial ovarian cancer progression by activating IGF2/IGF1R/PI3K/AKT pathway. So we guess that inhibited this pathway maybe a potential therapy target in ovarian cancer which elevated expression of TMED2.

MiR-30a suppresses tumor metastasis and proliferate in many cancers [21–23]. miR-30a can regulate malignant behavior in ovarian cancer [24]. miR-30a regulated PI3K/AKT pathway by directly target IGF1R in non-small cell lung cancer [25]. Our study also found that IGF1R is a directly target of miR-30a. So our finding is consistent with this report. We also determined that miR-30a regulated proliferate, migration and invasion through targeting TMED2. This data indicated that TMED2 was an tumor-promoting gene.

To sum up, this research found that TMED2 is a putative oncogene in ovarian cancer. It is related to IGF2/IGF1R/PI3K/AKT pathway. This implicated that TMED2 is a potential treatment target in epithelial ovarian carcinoma.

## MATERIALS AND METHODS

### Tissue specimens

The tissue microarray slides containing malignant and benign ovarian tissues (n=148) were obtained from US Biomax Inc cancer tissue bank collection (US Biomax Inc., MD, USA).

### Cell culture, transfection procedure, and reagents

The A2780 cell line was established from tumor tissue from an untreated patient. The ES-2 cell line was established from a surgical tumor specimen taken from a 47 year old black woman. SKOV3 human ovarian cancer cell line derived from the ascites from a 64 year old caucasian female with an ovarian tumor. The COC1 cell line was derived from the ascites of patients with poorly differentiated ovarian cancer. Cisplatin-resistant COC1/ DDP, which is derived from its parental ovarian cancer cell line COC1 by stepwise selection *in vitro* using cisplatin. CAOV-3 human ovarian cancer cell line derived from a 54 years old caucasian female with ovarian adenocarcinoma. Human ovarian cancer cells were cultured in Rosewall Park Memorial Institute (RPM1)1640 medium containing 10% fetal bovine serum and antibiotics, and incubated in an atmosphere with 5% carbon dioxide at 37°C. Double-strand oligonucleotides corresponding to the target sequences were synthesized by Genepharma Co., Ltd. (Shanghai, China). The following sequences were targeted for human TMED2, IGF1R, IGF2, PI3K and AKT2 small interfering RNA (siRNA), respectively. TMED2-1:5'-AGCUAGAAGAAAUGAUCAAUG-3'; TMED2-2:5'- GGUUCAAGUCACUAAAGAUUC-3'; IGF1R: 5'-GCGGAGAGAUGUCAUGCAAGU-3'; IGF2: 5'-CGUGUUUGACUCAACUCAACU-3'; IGF2: 5'-GCGGAGAGAUGUCAUGCAAGU-3'; PI3K: 5'-GGAUCAAGUUGUCAAAGAAGA-3'; AKT2: 5'-GGUUCUUCCUCAGCAUCAACU-3' and NC (negative control) siRNA: 5'-UUCUUCGAAGGUGUCACGUTT-3'. Lentiviral vector expressing shRNA targeting TMED2 (named LV3-1 and LV3-2) and TMED2-lentiviral expression vector (named LV5-TMED2) were provided from Genepharma Co., Ltd. (Shanghai, China). miR-30a mimics were synthesized at Ruibo Biotech (Ruibo Biotechnology, Guangzhou, China).

### Immunohistochemistry

Immunohistochemistry (IHC) was performed according to the SP kit instructions (SP-9000, ZSGB-BIO, Beijing, China). After dewaxing and hydration, the sections were heated in citrate buffer (pH 6.0, Sigma-Aldrich, USA) in a microwave oven for 20 minutes for antigen retrieval. Further, the sections were cooled naturally to room temperature. The sections were washed thrice for three minutes per cycle. Subsequently, the sections were incubated in 3% aquae hydrogenii dioxidi for 15 minutes at room temperature, washed thrice with phosphate buffered saline (PBS) for 3 minutes per cycles. The sections were blocked with 5% goat serum (ab7481; Abcam Company) for 30 minutes at 37°C. Anti-TMED2 rabbit polyclonal antibody (1:100, Sigma) was incubated with the sections overnight at 4°C. Negative controls included omission of primary antibody and use of irrelevant primary antibodies. The corresponding secondary antibodies that were conjugated to horseradish peroxidase (Bioss Biotechnology), were incubated with the sections for an hour at room temperature. The sections were washed thrice in PBS for 3 minutes per cycle. The sections were incubated in horseradish enzyme-labeled chain avidin solution (Bioss Biotechnology) for 30 minutes at 37°C, and washed in PBS for 3 minutes x 3 cycles. The proteins were visualized by diaminobenzidine. All the sections were observed by three independent pathologists using a light microscope. The staining data were obtained from manually recorded reports. Staining intensity was graded on a 0–3 scale as follows: 0 (absence of staining), 1 (weakly stained), 2 (moderately stained), and 3 (strongly stained). The percentage of positive tumor cells was scored as follows: 0 (absence of tumor cells), 1 (<33% tumor cells), 2 (33–66% tumor cells) and 3 (>66% tumor cells). The staining score was calculated as the product of staining intensity score and the percentage score; this score ranged from 0 to 9 (absence, IHC = 0; weak, 0< IHC ≤ 4; strong, 5 ≤ IHC ≤9) [26]

### F-actin staining

Ovarian cancer SKOV3 and A2780 cells were seeded (1×10^4^) on the cover slip and allowed to adhere overnight. Twenty-four hours after transfection, cells were fixed, permeabilized and stained with Tetramethylrhodamine (TRITC)-conjugated phalloidin (Sigma-Aldrich, Louis, USA) for 2 hour. Nuclei were stained with Hochest (Sigma-Aldrich, Louis, USA) for 15 min. The results were analyzed with fluorescence microscope.

### Quantitative real-time polymerase chain reaction(PCR)

Total RNA was isolated using a RNA pure High-purity Total RNA Rapid Extraction Kit (BioTeke, RP1201, China), as per the instructions provided in the kit. cDNA was synthesized using the iSCRIPT cDNA synthesis kit (Bio-Rad). The primers used for amplifying TMED2, IGF1R and GAPDH were synthesized by Guangzhou Funeng Co., Ltd. The real-time PCR kit was purchased from Guangzhou Funeng Co., Ltd. PCR conditions were 95°C for 10 seconds, 60°C for 20 seconds, 72°C for 10seconds. Each sample was analyzed in triplicate. Relative quantification of mRNA was performed using the comparative threshold cycles (CT) method. This value was used to plot the gene expression employing the formula 2^−Δ ΔCT^.

### Detection of protein expression by western blotting

Expression of TMED2, IGF1R, IGF2, PI3K, AKT and GAPDH protein were analyzed by the Western blot method, as described [27–29]. The primary antibodies used included polyclonal rabbit anti-TMED2 (1:1000; HPA014060; Sigma, USA); polyclonal rabbit anti-IGF1R (1:1000; ab39398; Abcam Inc., Cambridge, MA, USA); polyclonal rabbit anti-IGF2 (1:1000; ab9574; Abcam Inc., Cambridge, MA, USA); anti-PI3K antibody rabbit polyclonal antibody (1:1000, ab86714, Abcam Inc., Cambridge, MA, USA); anti-AKT2 antibody rabbit polyclonal antibody (1:1000, ab175354, Abcam Inc., Cambridge, MA, USA) and polyclonal rabbit anti-GAPDH (1:1000; ab8245; Abcam Inc., Cambridge, MA, USA). The band density was analyzed using a gel imaging system and compared against an internal control.

### Cell proliferation assay

Cell proliferation was determined using the EdU assay was performed using the Cell-Light TM EdU imaging detecting kit according to the instructions in the kit (Ruibo Biotechnology, Guangzhou, China). EdU is a thymidine analog that can be used to label cells undergoing DNA replication [30–32]. Cell proliferation was assessed by the CCK-8 assay (Beyotime, Shanghai, China, C0037) following to the manufacture's instruction. Cells were seeded into 96-well plates and cultured for an additional 24 h. After treatment with OT, 10 μl of the kit reagent was added and then incubated for another 2 h. O.D. value was read at 450 nm to obtain the final results [33–35].

### Wound healing assay and matrigel invasion assays

Migration of SKOV3 and A2780 cells was analyzed using the wound-healing assay *in vitro*. Cells were seeded into 6-well plates and cultivated until 90% growth confluence. Wounds were afflicted by scraping the monolayer cells with a sterile pipette tip. At 0, 24, 48 and 72 hours after the wounding, cells were observed under low power in an Olympus light microscope. The distance between two wounds were measured at each time point, and expressed as the average percent of wound closure as compared to that at zero time. Invasion of SKOV3 and A2780 cells was evaluated by Matrigel invasion assays. For Transwell invasion assays, the upper side of an 8 μm pore, 6.5-mm polycarbonate transwell filter (Corning, New York, NY) chamber was uniformly coated with Matrigel basement membrane matrix (BD Biosciences, Bedford, MA) for 2 h at 37°C before cells were added. A total of 5 × 104 cells were seeded into the top chamber of a trans-well filter (in triplicate) and incubated for 48 hours. Invasive cells on the lower side of the filter, were fixed in 4% paraformaldehyde, stained in 0.5% crystal violet (Beyotime), and counted using a microscope. A total of five fields were counted for each transwell filter. Each field was counted and photographed at 200 × magnification.

### *In vivo* tumor xenograft study

All procedures for animal experiments were approved by the Committee on the Use and Care on Animals (Southern Medical University, Guanzhou, China), and performed in accordance with the institution guidelines. Ovarian cancer SKOV3 cells were infected with indicated lentiviral vectors and injected (1×10^6^ cells per mouse in 200 ul) subcutaneously into the left armpit of 6-week-old BALB/c nude mice (LV3-NC infected group, n =5; LV3-1 infected group, n=5). Twenty eight days later, animals were sacrificed to confirm the presence of tumors, and weigh the established tumors.

### Statistical analysis

All statistical analyses were performed using SPSS software, version 17.0 (Chicago, IL). Each experiment was performed in triplicate. Statistical analysis was performed by Student’s t-test or analysis of variance (ANOVA). Data were presented as Mean ± standard deviation. Statistical significance was defined as a p-value less than 0.05.

## Abbreviations

TMED: transmembrane emp24 domain
IGF2: insulin-like growth factor-2
IGF1R: type 1 insulin-like growth factor receptor
PI3K: phosphatidylinositide 3-kinase
miR-30a: microRNA 30a
LV3-NC: LV3-shTMED2-negative control
LV3-1: LV3-shTMED2-1
LV3-2: LV3-shTMED2-2
